# Predictive Values of the CHA2DS2-VASc Score and Left Atrial Diameter for Cerebral Small Vessel Disease in Geriatric Patients With Atrial Fibrillation

**DOI:** 10.7759/cureus.47764

**Published:** 2023-10-26

**Authors:** Anıl Tanburoglu, Ismail Karluka, Sevda Diker, Pınar Gelener

**Affiliations:** 1 Neurology, Başkent University, Adana, TUR; 2 Radiology, Faculty of Medicine, Başkent University, Adana, TUR; 3 Neurology, Cyprus International University, Nicosia, CYP; 4 Neurology, Faculty of Medicine, University of Kyrenia, Kyrenia, CYP

**Keywords:** geriatric age, atrial fibrillation, left atrial diameter (lad), white matter hyperintensity, stroke, small vessel disease

## Abstract

Objective

The objective of this study was to determine whether the CHA2DS2-VASc (congestive heart failure, hypertension, age, diabetes mellitus, stroke, vascular disease, age, sex) score and left atrial diameter (LAD) could predict the presence of cerebral small vessel disease (cSVD) in patients older than 65 years with atrial fibrillation as the cause of ischemic stroke.

Materials and methods

In this study, we included patients over 65 years of age who had suffered an ischemic stroke caused by atrial fibrillation within 30 days after the onset of symptoms. The data recorded included demographics, electrocardiograms, Holter monitors, and echocardiography reports. The anteroposterior LAD, determined by transthoracic echocardiography, was analyzed. Each patient's CHA2DS2-VASc score was calculated. Brain magnetic resonance imaging (MRI) assessed white matter hyperintensities (WMH) on fluid-attenuated inversion recovery (FLAIR) images and cerebral microbleeds (CMBs) on susceptibility-weighted sequences. The Fazekas score, based on WMH on MRI, was used to grade the severity of gliosis. Participants were categorized into three groups according to their quantitative CMB burden.

Findings

The study included 60 participants, with a mean age of 80 years (range 65-99), and 43.3% (n = 26) were male. The CHA2DS2-VASc score had a mean value of 4.21 (range 2-8), and the mean LAD was 4.17 (range 2.6-5.3) cm. The CHA2DS2-VASc score did not predict CMBs (OR, 1.389; 95% CI, 0.961-2.008, p = 0.08) in geriatric stroke patients with atrial fibrillation. However, in the subgroup of patients with diabetes mellitus, the CHA2DS2-VASc score was higher in those with CMB 1-4 and CMB ≥ 5 than in those without CMB. Additionally, the risk of CMBs 1-4 increased with higher LAD compared to patients without LAD.

Conclusion

The LAD and CHA2DS2-VASc scores were not significantly associated with CMB prediction in elderly stroke patients with atrial fibrillation. In a diabetes mellitus subgroup, the CHA2DS2-VASc score was indicative of CMB. An increased LAD elevates the risk of CMBs in patients with coronary artery disease.

## Introduction

Atrial fibrillation (AF) and cerebral small vessel disease (cSVD) are two common conditions that increase the risk of stroke in the elderly population [1,2]. AF is associated with an increased risk of mortality, stroke, thromboembolism, and heart failure [2]. It leads to a fivefold increase in the risk of ischemic stroke [3]. Both an increment in the CHA2DS2-VASc (congestive heart failure, hypertension, age, diabetes mellitus, stroke, vascular disease, age, sex) score and left atrial enlargement potentiate the risk of cardioembolic stroke in patients with AF [4-5]. The CHA2DS2-VASc score is calculated based on age and vascular risk factors such as hypertension, diabetes, prior stroke, and vascular disease. The size of the left atrium is influenced by factors like pressure in the left ventricle during filling, heart function during relaxation, and the regulation of blood flow [6]. An enlarged left atrium is associated with higher mortality and cardiovascular events, including stroke [7].

On the other hand, cSVD affects small arteries, arterioles, venules, and capillaries in the brain. The underlying etiologies include arteriolosclerosis due to aging, hypertension, other vascular risk factors, and vascular beta-amyloid accumulation. Magnetic resonance imaging (MRI) findings associated with cSVD include deep and periventricular white matter hyperintensities (WMHs), cerebral microbleeds (CMBs), lacunes, and enlarged perivascular spaces. Its prevalence ranges from 3 to 34% for CMBs and 39-96% for WMHs in older adults [8-10]. Specifically, WMHs are a significant risk factor for dementia, stroke, gait instability, and depression. WMHs are associated with worse outcomes in stroke patients [11-12].

This study aims to investigate the potential value of clinical parameters, such as the CHA2DS2-VASc score and left atrial diameter (LAD), in predicting the risk of cSVD. The efficacy of these parameters in forecasting cSVD can play a critical role in the early diagnosis and treatment of individuals at risk. This could guide more aggressive clinical approaches with the potential to slow or halt the progression of cSVD.

This study was presented at the 3rd International 14th Academic Geriatrics Congress in 2021 in Turkey as a meeting abstract.

## Materials and methods

All consecutive patients admitted to our university hospital's emergency and neurology departments with ischemic stroke or transient ischemic attack (TIA) between January 2019 and July 2021 were retrospectively reviewed using patient files and the center's database. Patients were enrolled if they were 65 years old or older and had a history of AF or had an ECG or Holter recording showing AF during their current admission.

Participants with contraindications to MRI, including intracardiac defibrillators or pacemakers, metallic heart valves, severe renal or hepatic insufficiency, mitral stenosis, and previous heart valve surgery, were excluded [13]. Patient demographics, history of diabetes mellitus, hypertension, hyperlipidemia, symptom duration at admission, electrocardiographic findings, and Holter rhythm were recorded.

Transthoracic echocardiography findings were obtained, including ejection fraction and anteroposterior left atrium diameter. The left atrial dimension at end-systole and LV ejection fraction were obtained according to the American Society of Echocardiography standards [14].

Among the risk stratification tools, the CHA2DS2-VASc score is widely used to predict the future risk of ischemic stroke in patients with nonvalvular AF. The CHA2DS2-VASc score (congestive heart failure, hypertension, age 75 years or older (doubled), diabetes mellitus, prior stroke or TIA (doubled), vascular disease, age 65-74 years, female) was calculated for each patient [4]. When scoring for a prior stroke or TIA, the current event on admission was not included.

Patients with hypertension were considered present if they had a systolic blood pressure greater than 140 mm Hg or a diastolic blood pressure greater than 90 mm Hg before the stroke, or if they had a documented history of hypertension in addition to using antihypertensive medications. Diabetes mellitus (DM) was considered present if participants had a history of using any type of antidiabetic medication or if their fasting blood glucose exceeded 126 mg/dl. Hyperlipidemia was considered present if the individual was taking lipid-lowering drugs or had a total cholesterol level exceeding 220 mg/dl or an LDL cholesterol level surpassing 130 mg/dl.

MRI scans of the brain were examined using the picture archiving and communication system (PACS) in the radiology department. Radiologists retrospectively assessed images from the T2 series, fluid-attenuated recovery (FLAIR), diffusion-weighted imaging (DWI), apparent diffusion coefficient map, and susceptibility-weighted imaging (SWI) sequences acquired on the 1.5T MR system (Magnetom Aera 1.5T, Siemens Healthcare, Erlangen, Germany). The evaluation was conducted without knowledge of clinical information. CMBs were defined as signal void spots with ovoid or rounded shapes measuring less than 1 cm in the T2* GRE or SWI sequences. Signal voids resulting from sulcal vascular structures, old hemorrhagic cerebrovascular event sequelae, basal ganglia calcifications, or pineal gland calcifications were excluded.

WMHs were assessed utilizing FLAIR images incorporated into the MRI protocol. Gliosis severity was determined through the Fazekas score, which grades white matter hyperintensities on MRI. The degree of WMH severity was categorized on a scale: 0 = absent, 1 = punctuate foci, 2 = the beginning of a confluence of foci, and 3 = large confluent areas.

Statistical analysis was performed using IBM SPSS Statistics for Windows, Version 25 (Released 2017; IBM Corp., Armonk, New York). A p-value of ≤0.05 was considered indicative of statistical significance. The Kolmogorov-Smirnov test was used to assess the distributions of continuous variables, determining whether each variable followed a normal distribution. Since the LAD and CHA2DS2-VASc variables did not adhere to a normal distribution, they were reported as median (minimum-maximum) values, while the age variable was reported as mean ± standard deviation.

Binary and multinomial backward stepwise logistic regression analyses were performed to determine the factors associated with CMBs and Fazekas types. These analyses were based on the LAD and the CHA2DS2-VASc score. The models were adjusted for gender, hypertension, diabetes mellitus, hyperlipidemia, coronary artery disease (CAD), TIA, or ischemic stroke. The assumptions of binary and multinomial logistic regression analyses were checked. The composed models were unaffected by the multicollinearity problem, and logistic regression analyses were satisfied. The logistic regression analysis results were reported in terms of odds ratio (OR) and 95% confidence intervals (CI) for the model's parameters.

This study was approved by the institutional review board of Baskent University (approval number: KA23/106).

## Results

Our study included 60 patients. The demographic and clinical characteristics of the patients are summarized in Table 1. The mean age was 77.9 ± 7.16 years (range 65-99), and 56.7% (n=34) of the patients were female. Hypertension was the most prevalent cardiovascular risk factor, affecting 78% (n=47) of the cohort. Approximately one-third of the patients had experienced a previous TIA or stroke. Nearly half of the patients, 46.5% (n=28) with SWI, had CMBs. A total of 71.7% (n=33) had moderate to severe WMH. Except for two patients with intermediate-risk CHA2DS2-VASc scores (1 in men or 2 in women), all had high-risk CHA2DS2-VASc scores of 2 points or more.

**Table 1:** Demographic and clinical characteristics of study patients TIA: transient ischemic attack, SWi: susceptibility-weighted imaging.

Characteristics	Value
Number of participants	60
Age, mean ± standard deviation, years	77.9 ± 7.164
Female sex, n (%)	34 (56.7)
Hypertension, n (%)	47 (78.3)
Diabetes mellitus, n (%)	12 (20)
Hyperlipidemia, n (%)	14 (23.3)
Coronary artery disease, n (%)	15 (25)
Previous TIA/ischemic stroke, n (%)	19 (31.7)
Fazekas score 0 and 1, n (%)	17 (28.3)
Fazekas score 2 and 3, n (%)	43 (71.7)
Cerebral microbleed count, n (%): 0 SWI lacking	31 (51.7)
Cerebral microbleed count, n (%): 1-4 SWI lacking	27 (45)
Cerebral microbleed count, n (%): ≥5 SWI lacking	2 (3.3)
Left atrium diameter, median (Min-Max), cm	4 (2.6-5.3)
CHA2DS2-VASc score, median (Min-Max)	4 (2-8)

There was no statistically significant association between LAD and CHA2DS2-VASc score (p=0.142). 

The CHA2DS2-VASc score was not predictive of the presence of CMBs (OR, 1.389; 95% CI, 0.961-2.008; p=0.08) in geriatric stroke patients with AF. However, it was predictive for CMB 1-4 and CMB ≥ 5 in a subgroup of patients with DM (Table 2). Moderate to severe WMH did not have a predictive value (OR, 95% CI: 1.305, 0.895-1.902; p=0.167).

**Table 2:** Logistic regression analysis for cSVD based on the CHA2DS2-VASc score OR: odds ratio, CI: confidence interval, cSVD: cerebral small vessel disease, DM: diabetes mellitus, CMB: cerebral microbleed. p-value of ≤0.05 was considered indicative of statistical significance (95% CI).

Models		OR (95% CI)	p-Value	Nagelkerke R square
Model I	CMB ³ 1 vs 0	1.389 (0.961-2.008)	0.08	0.074
Model II	CMB 1-4 vs 0			0.311
	CHA2DS2-VASc	1.013 (0.742-1.381)	0.937	
	DM = 0* CHA2DS2-VASc	0.646 (0.432-0.966)	0.033	
	CMB ³ 5 vs 0			
	CHA2DS2-VASc	1.170 (0.898-1.524)	0.244	
	DM = 0* CHA2DS2-VASc	0.730 (0.537-0.993)	0.045	
Model III	Fazekas 2-3 vs 0-1	1.305 (0.895-1.902)	0.167	0.055

The CHA2DS2-VASc score did not predict the overall presence of CMBs in geriatric stroke patients with AF. The OR was 1.389 with a 95% CI of 0.961-2.008, and the p-value was 0.08, indicating no statistically significant association.

However, in a subgroup of patients with diabetes mellitus (DM), the CHA2DS2-VASc score was predictive for (1) CMB counts between 1 and 4 and (2) CMB counts of 5 or more. The specific odds ratios and confidence intervals for these associations are detailed in Table 2.

Moderate to severe WMHs did not have predictive value for cerebral microbleeds (CMBs). The odds ratio was 1.305 with a 95% confidence interval of 0.895-1.902, and the p-value was 0.167, indicating no statistically significant association.

This suggests that although the CHA2DS2-VASc score may not predict CMBs in the general population of geriatric stroke patients with AF, it could have predictive value in a specific subgroup of these patients with diabetes mellitus. Additionally, moderate to severe WMHs did not predict CMBs in this study.

LAD was not found to be a predictive factor for the presence of cerebral microbleeds (CMBs) or for moderate to severe WMHs (OR, 95% CI: 0.595, 0.225-1.570, p=0.294). Statistical analyses indicated no significant association between LAD and these two conditions. However, a more in-depth regression analysis revealed a different outcome for patients with CAD. According to this analysis, in patients with CAD, the risk of having 1-4 CMBs increased as the LAD value increased. This finding suggests a higher risk compared to patients without CAD. These relationships and findings are detailed further in the tables referenced in the study (Table 3).

**Table 3:** Logistic regression analysis for cSVD based on LAD OR: odds ratio, CI: confidence interval, cSVD: cerebral small vessel disease, LAD: left atrial diameter, CAD: coronary artery disease, CMB: cerebral microbleed. p-value of ≤0.05 was considered indicative of statistical significance (95% CI).

Models		OR (95% CI)	p-value	Nagelkerke R square
Model IV	CMB ≥1 vs 0	1.322 (0.580-3.016)	0.507	0.013
Model V	CMB 1-4 vs 0			0.264
	CAD=0*LAD	0.767 (0.610-0.963)	0.023	
	CAD=1*LAD	0.640 (0.394-1.039)	0.071	
	CMB ≥ 5 vs 0			
	CAD=0*LAD	0.844 (0.693-1.027)	0.091	
	CAD=1*LAD	0.965 (0.755-1.233)	0.774	
Model VI	Fazekas 2-3 vs 0-1	0.595 (0.225-1.570)	0.294	0.035

This summary highlights that while LAD is not a general predictor, it may be associated with the risk of CMBs in a specific patient group (those with CAD).

## Discussion

CMB is associated with an increased risk of both ischemic and hemorrhagic strokes. Their microhemorrhagic nature has been linked to increased hemorrhagic transformation following acute stroke [15]. Moreover, they influence individualized therapy when deciding on antiplatelet or anticoagulant therapy for patients suffering from cerebrovascular disorders [16]. AF and older age are associated with increased CMB load, making the prevalence of CMB high (46.5%) in our cohort [17,18]. In the Rotterdam Scan Study, the prevalence of CMB increased with age, from 6% in those aged 45-50 years to 36% in those aged 80 or older [19]. When risk factors for CMB, including age, hypertension, previous TIA/stroke, and AF are considered, an association between CHA2DS2-VASc score and CMB load is expected [17-21]. Our study found that the CHA2DS2-VASc score predicted a high (≥ 5) CMB load in geriatric stroke patients with AF. Similarly, in the literature, it has been shown that the CHA2DS2-VASc score can predict the presence and severity of CMB in nonvalvular AF patients with stroke [22].

WMH is linked to an increased risk of stroke, cognitive decline, dementia, and mortality. In most older individuals, the underlying pathology of WMH is chronic ischemia [23]. According to population-based studies, the main risk factors for developing white matter lesions are greater age, arterial hypertension, and cardiovascular disease [10,24]. The prevalence of any WMH in the general population ranges from 39% to 96%. In the population-based Cardiovascular Health Study and Rotterdam Scan Study, which only included participants above 60 years of age, the prevalence of any WMHs was as high as 96% and 95%, respectively [25-27].

There is no gold standard for assessing WMHs. Methods for quantifying the presence and degree of severity of these lesions range from visual rating to automated methods. Visual rating scales save time and are highly reliable if the rater is experienced. In addition, sophisticated and expensive post-processing facilities are not needed. Automated methods provide precise WMH volumes, making them advantageous [10]. One widely used visual rating scale is the Fazekas rating scale, used in our study [28].

Although there was a trend toward increased Fazekas score with increasing CHA2DS2-VASc score, this did not reach statistical significance in our study. All items in the CHA2DS2-VASc score analysis can directly or indirectly relate to WMH pathogenesis. As expected, studies show a positive correlation between CHA2DS2-VASc score and leukoaraiosis severity. In a study among nonvalvular AF patients aged 65 years or older, total WMH lesion volume was higher in those with increased risk of CHA2DS2-VASc score (n=40) than in those with intermediate risk (score of 1 in men or 2 in women) of CHA2DS2-VASc score (n=9) [29]. Using the Fazekas scoring system instead of volumetric measures for WMH may be partly the reason for our not finding an association.

Regarding the LAD, we could not find a predictive value for WMH. In a study investigating the association between LAD and vascular brain injury on brain MRI among community-dwelling adults aged 65 years or older, participants were evaluated by follow-up MRI approximately five years later [30]. The LAD was independently associated with prevalent brain infarcts, particularly non-lacunar infarcts, but not with leukoaraiosis or worsening leukoaraiosis over time. CMB was not analyzed in the study. The correlation between the LAD and the CMB severity has not been sufficiently investigated. Our study demonstrated that in geriatric ischemic stroke patients with AF and CAD, the risk of CMB between 1 and 4 increased as LAD increased. Prospective studies with a more substantial number of participants are required in this field.

Our study has some limitations. It was a retrospective study conducted in a single center with a relatively small sample size. Volumetric measurements of brain WMH or CMB load and left atrial volume were not performed. However, we included a specific population of geriatric patients with ischemic stroke and AF. While the association of CHA2DS2-VASc score with cSVD findings has been analyzed extensively, this was not the case for LAD and radiological correlations. The strength of our study is the extensive radiological evaluation, including SWI sequence and investigating the effects of CHA2DS2-VASc score and LAD.

## Conclusions

In the geriatric population, AF and cSVD are both commonly occurring conditions with significant implications for stroke risk and overall morbidity. Our study aimed to assess whether the CHA2DS2-VASc score and LAD could serve as predictive markers for cSVD manifestations. In specific subgroups, the CHA2DS2-VASc score demonstrated some predictive capacity for CMBs, but it was not statistically significant for WMH. Similarly, LAD did not show a significant predictive value for CMB or WMH, although certain correlations were observed in CAD patients.

It is evident from the findings that the relationship between cardiac parameters and cerebral vascular changes is complex. While the CHA2DS2-VASc score and LAD provide valuable insights into cardiovascular risk, their direct applicability as predictors of cSVD requires further investigation. The study's limitations, such as its retrospective nature and the absence of volumetric measurements, highlight areas for future research.

Our study sheds light on some associations between AF-related parameters and cSVD. However, it also emphasizes the need for more comprehensive, prospective studies to validate and expand upon these findings. A study of this nature could lead to more accurate risk stratification and targeted interventions designed to mitigate the impact of cSVD in elderly patients with AF.
